# The effect of adjunctive LASER application on periodontal ligament stem cells

**DOI:** 10.3389/fcell.2023.1341628

**Published:** 2024-01-12

**Authors:** Deepa Ponnaiyan, Roshan R. Rughwani, Ganesh Shetty, Jaideep Mahendra

**Affiliations:** ^1^ SRM Dental College, Chennai, Tamil Nadu, India; ^2^ Namma Dentist Dental Clinic, Chennai, India; ^3^ Dental and Orthodontic Clinic, Bangalore, India; ^4^ Meenakshi Academy of Higher Education and Research, Chennai, Tamil Nadu, India

**Keywords:** PDLSCs (periodontal ligament stem cells), regeneration, periodontal regeneration, stem cell, dental stem cell, photobiomodulation, laser

## Abstract

Periodontal regeneration involves the composite action of cell, scaffolds and signaling molecules. There are numerous autologous sources of regenerative cells which are present close to the vicinity of the periodontally debilitated site, the primary one being the periodontal ligament stem cell, which is believed to have a key role in regeneration. Various methods can be harnessed to optimize and enhance the regenerative potential of PDLSCs such as the application of LASERs. In the last few years there have been various studies which have evaluated the effect of different types of LASERs on PDLSCs and the present review summarizes the photo-biomodulative activity of LASERs in general and its beneficial role in the stimulation of PDLSC specifically.

## Introduction

Periodontitis is a disease primarily characterized by inflammation and an interplay of bacteria and endotoxins which impacts the soft and hard tissues of the periodontium. The disease leads to significant cellular damage and tissue loss which eventually culminates in bone loss (Hajishengallis, 2022; Zenobia and Darveau, 2022; Vitkov et al., 2023). The extent of hard and soft tissue loss determines the treatment strategies; however, mechanical debridement remains the cornerstone of periodontal treatment which turns the inflammatory state of the periodontium to a resolving state (Albeshri and Greenstein, 2022; Laleman et al., 2022). Periodontal treatment not only involves elimination of inflammatory and bacterial component from the diseased tooth supporting tissues, but also comprises of regeneration of deteriorated periodontal structures in amenable cases which serves as the bedrock to principles of tissue engineering that engages application of appropriate cells, growth factors and scaffolds Tavelli et al. (2022), Yi et al. (2022), Sopi et al. (2023).

Dental stem cells owing to their distinct stemness, migration, differentiation and immunological modulation properties, have been perceived as a potential agent for regeneration (Nagata et al., 2022; Sun et al., 2023). Stomatognathic stem cells are placed in different niches and they can be further categorized as dental and periodontal stem cells based on their location within the oral complex (Ponnaiyan et al., 2022; Alarcón-Apablaza et al., 2023). Recently, research shows that periodontal ligament stem cells that are mesenchymal in nature and located within the periodontal ligament, offers substantial periodontal regeneration in comparison to the regenerative outputs offered by other kinds of stem cells (Li et al., 2020; Dubuc et al., 2022; Mendoza et al., 2023).

The regenerative output of PDLSCs is further enhanced by the adjuvant application of LASERs as evidenced by literature (Mylona et al., 2022; L; Wang et al., 2022). Hence, the current review aims to summarize the additional use of LASERs in regenerative and stem cell therapy in periodontics.

## Periodontal ligament stem cells (PDLSCs)

PDLSCs are formed from ecto-mesenchymal cells that originate from the neural crest and are isolated from middle third of the root surface after extraction of permanent teeth (T. Wang et al., 2022). Root surface derived PDLSCs (r-PDLSCs) are stem cells isolated from root surface, while alveolar socket derived PDLSCs (a-PDLSCs) are stem cells separated from tissue taken from the bone surface (Rad et al., 2022). In comparison to r-PDLSCs, it has been found that a-PDLSCs maintain a higher proliferative capability as well as strong osteogenic and adipogenic potential (Nagata et al., 2022). PDLSCs can differentiate into peripheral nerves, blood vessels, alveolar bone, cementum and even periodontal ligament (Seo et al., 2004). It is interesting to know that PDLSCs phenotypically express various neural crest, embryonic and antigenic markers which contribute to its diverse multipotent differentiation characteristics (Iwayama et al., 2022). They express standard mesenchymal stem cells markers and are negative for hematopoietic markers (Song et al., 2023). Amongst embryonic markers they highly express Nanog, SRY (sex determining region Y)-box 2, also known as SOX2, SSEA4 (Stage specific embryonic Antigen) and express October 4 (Octamer) and Krüppel-like factor 4 (KLF4) in lower levels (Alves et al., 2023; Takahashi et al., 2023). However, the expression levels of these markers are highly dependent on the environment in which the PDLSC is residing as inflammation affects stem cells and those cells in turn have immune-modulatory effect in an inflammatory milieu (Zhang et al., 2021; Liu et al., 2022).

The equilibrium between the pro-inflammatory response and the stemness of PDLSCs is a key factor in determining whether tissue integrity and homeostasis is maintained or if disease progression occurs (Zhao et al., 2022). It has been observed that long-term stimulation of PDLSCs by P. gingivalis lipo-polysaccharide (LPS) resulted in an increase in cellular cytokine production and LPS also prevents the development of osteoblasts by impairing ALP activity and mineral synthesis in PDLSCs (Chen et al., 2021; Xu et al., 2023).

Conversely PDLSCs also have an immunomodulatory influence on periodontal regeneration (Andrukhov et al., 2019; Liu et al., 2022). PDLSCs also affect the innate immune response by increasing neutrophil proliferation and decreasing their capacity to undergo apoptosis. PDLSCs also promote CD-136, IL-10, and Arginase 1, which enhances the anti-inflammatory M2 macrophage phenotypic polarization in addition to inhibiting T cell proliferation (Shin et al., 2017; Li et al., 2022). These evidences suggest that, to harness the maximum regenerative potential of PDLSCs, it is imperative to maintain the state of controlled inflammation or homeostasis which favors repair which is possible only through the elimination of the pro-inflammatory components, bacteria and endotoxins via mechanical debridement thereby, declaring LASERs to be an adjunct to the mechanical therapy (Jiang et al., 2022; Lu et al., 2023).

## LASERs

The LASER revolution in dentistry and the invention of ruby LASER by Theodore Maiman in 1960 has seen significant changes over the past few decades owing to advances and usability of LASERs in a multitude of dental and periodontal applications (Passanezi et al., 2015; Theodoro et al., 2021; Corbella et al., 2023). LASERs are clinically categorized into two types based on their wavelength: 1) a deeply penetrating type where the LASER light penetrates and scatters into the tissue deeply, such as the diode LASERs (810–980 nm) and neodymium-doped yttrium-aluminum-garnet (Nd:YAG) available for clinical application; and 2) a superficially absorbed type such are the erbium-doped yttrium-aluminum-garnet (Er:YAG) (2940 nm), carbon dioxide (CO_2_) (10,600 nm) and erbium, chromium: yttrium-scandium-gallium-garnet (Er, Cr:YSGG) (2780 nm) LASERs (Ohsugi et al., 2020; Fu and Wo, 2021).

When LASERs energy penetrates the tissue surface, it may be absorbed, dispersed, reflected, or transmitted to the cells in the vicinity and the amount of energy that is absorbed by the cells influences the therapeutic and photo-biomodulative property exhibited by the LASER (Glass, 2021; Lopes et al., 2022). Since the 20th Century, the photobiomodulative property of LASER has been discovered and this property has been harnessed for specific effect of LASERs on tissues and cells for achieving desired treatment outcomes (Arjmand et al., 2021). Photobiomodulation makes use of nonionizing light sources such as LASERs, light-emitting diodes and broad-spectrum light to encourage physiological changes and therapeutic effects which supra-adds to the stimulation of stem cells involved in the regeneration triad (Firoozi et al., 2022). The PBM property of LASER encompasses a wide array of properties such as the photothermal, photodynamic, bio stimulative, photo ablative, photo vaporolytic and photo plasmolytic properties which may alter the cellular dynamics of periodontal tissues when applied at different settings and for varying time periods (Glass, 2021; Parker et al., 2022).

Alternate and more compliant sources of light such as LASER and light-emitting diode (LED) sources emit a wide range of visible and infrared spectrum that are used in photo-biomodulation (PBM), which can be effectively used in the treatment of a number of diseases, wounds, and disorders (Dompe et al., 2020; Bunch, 2023). A well-accepted theory holds that the light energy given to tissues is absorbed by the cell chromophores, encouraging the generation of adenosine triphosphate (ATP) (Glass, 2021). Understanding the processes behind the effects of PBM has been of significant interest. Nevertheless, some researchers have observed favorable effect of ATP generation on oxidative stress, survival, and tissue regeneration (da Silva et al., 2023; Prado et al., 2023).

## Effect of LASER on PDLSCs

The effect of LASERs in periodontal regeneration is well established. Initially, various studies were done in the past to identify the effects of LASERs on native PDL cells. Studies have showed that stretched periodontal ligament cells during orthodontic treatment demonstrated marked reduction in the level of pro-inflammatory mediators on the application of diode LASERs (Ozawa et al., 1997; Yamauchi et al., 2018).

**FIGURE 1:**
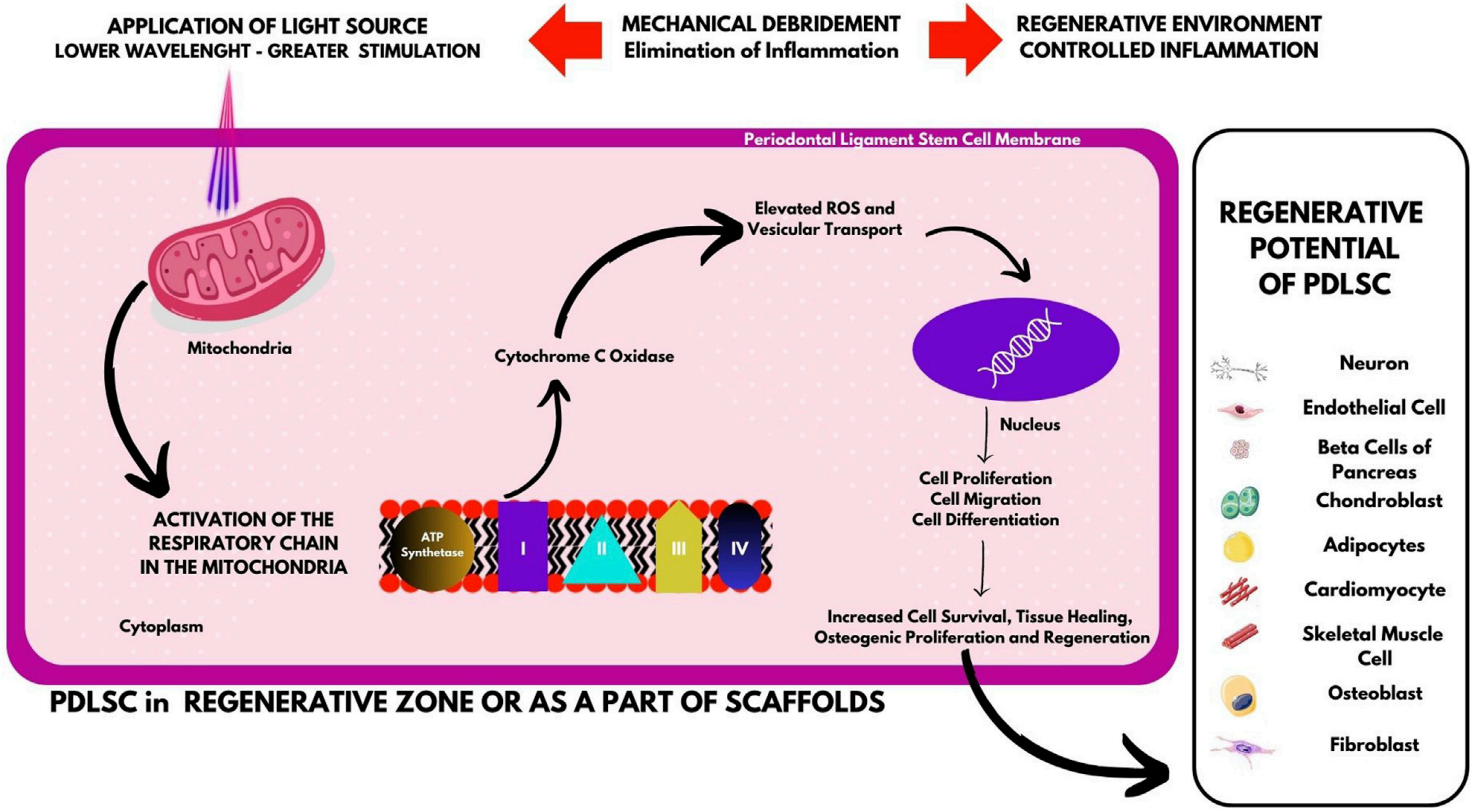
Effects of LASER at the cellular level on the stimulation of PDLSCs.

With advances in orthodontic research, it is understood that tooth movement was a PDL phenomenon which occurred as a result of coupled resorptive and regenerative activities of the periodontium (Li et al., 2021). With the advent of stem cell isolation techniques, it was later seen that not only fully differentiated PDL cells but also these stem cells residing in the periodontal ligament, took part in regeneration (Costela-Ruiz et al., 2022). As it is known that regeneration involves the Melcher’s triad, the cells and in particular, the stem cells become an indispensable tool to achieve optimal regeneration (Ward, 2022; Yi et al., 2022). To expedite the regenerative potential of these stem cells, various light sources including LASERs and LEDs have been proven to be beneficial in the stimulation of the PDLSCs as mentioned in Table 1. It is interesting to know that the maximal beneficial role of PDLSCs can be achieved only at certain irradiation settings, thereby making the wavelength, irradiation time interval and the energy setting to be the key regulating factors in optimal stimulation of PDLSCs by light sources (Oyebode and Houreld, 2022; da Silva et al., 2023). LASER application causes the release of a photoreceptor called cyctochrome-c oxidase (CCO), which is found in the mitochondrial respiratory chain at unit IV. This raises the potential of the mitochondrial membrane and produces more ATP, which in turn causes cell division (Signaling in Photobiomodulation, 2018; Pan et al., 2022) (Figure 1).

**TABLE 1:** Effect of different types of LASERs on PDLSCs.

LASER type	Irradiation protocol	Outcome	Reference/Author
Gallium-aluminum-arsenide (GaAlAs) 660 nm red LASER	Power—15.17 mW/cm^2^, Distance 3cm, Fluences at 0, 1, 2, 4 J/cm^2^	Proliferation	Wu et al. (2013)
	Exposure time—66, 132 and 264 s respectively	3rd day: 2 J/cm^2^ significantly better	
		5th day: 1 and 2 J/cm^2^ significantly better	
		Osteogenesis: Progressive significant increase at 7, 10, 14 days for 2 and 4 J/cm^2^ compared to control	
Indium-Gallium-Aluminum-Phosphide (InGaAIP) diode LASER 660 nm	30 mW,0.5 and 1.0 J/cm^2^	Proliferation: The group that received a dose of 1.0 J/cm^2^ boosted cell proliferation after 48 and 72 h *versus* the other two groups	Soares et al. (2015)
He-Ne LASER 632.8 nm	20 J/cm^2^, HF-LPLI for 1 h	Proliferation: Improved for cells cultured 6 h followed by LASER irradiation	Hou et al. (2018)
		Osteogenic differentiation: Better for cells cultured after 6 h followed by LASER irradiation	
LED 600–700 nm	Total irradiance 200 mW/cm^2^, CW mode, distance 40mm, fluences at 1, 2, 4, 6, 8, or 10 J/cm^2^	Proliferation: 1st day- 8 J/cm^2^ significantly better	Yamauchi et al. (2018)
		3rd day—4,6, 8 and 10 J/cm^2^ significantly better	
		But 8 J/cm^2^ highest	
		Osteogenesis: Increased at 21st day with 8 J/cm^2^	
Diode LASER 808 nm	100mW, spot area 0.5 cm^2^, fluences at 1 and 2 J/cm^2^, 2 sessions (0 and 48 h)	Proliferation: 7,14 and 21 days all groups better than control	Abdelgawad et al. (2020)
		LASER irradiation at 2 J/cm^2^ and Vit D increased differentiation and proliferation of PDLSCs into osteoblasts	
Near-infrared low-intensity diode LASER PBM -940 nm	Energy density of 4 J cm^2^ in a 100 mW continuous wave	Proliferation: No significant difference between LASER and control group on 3rd day	Gholami et al. (2022b)
		Osteogenesis: Slight significant increase in osteogenic gene expression between LASER and control on 14th and 21st day	
low-energy red LED irradiation (600–700 nm)	CW, 2 cm distance	Proliferation: PDLSCs in the irradiation groups proliferated more than those in control group	Wu et al. (2021)
	66.7 mW/cm^2^, 1 J/cm^2^ for 15 s, 3 J/cm^2^ for 45 s	Osteogenesis	
	5 J/cm^2^ for 75 s	Increase in ALP seen only for 5 J/cm^2^ at day 7th, following which irradiation did not increase osteogenesis	
LEDs	3.5 J/cm2, CW, 20 min per day, 52 mm distance	Proliferation	Chaweewannakorn et al. (2021)
		Day 6th and 8th—830 nm significantly better than other groups	
		Day 8th—630–680 nm significantly worse than control	
		Osteogenesis	
		3rd day: 680 significantly better	
		7th day: 630 and 680 significantly better. 10th day: 630, 680, 830 significantly better. 14th day: 680 nm significantly better	
		21st and 28th day: both 630nm and 680 nm significantly better	
Diode LASER 808 nm	100 mW, CW, spot area 0.5 cm^2^, fluences of 1, 2 and 3 J/cm^2^	Proliferation	Mohamed Abdelgawad et al. (2021)
		1st day:LASER at 2 J/cm^2^, 3 J/cm^2^, met +1 J/cm^2^, met + 2J/cm^2^, met +3 J/cm^2^ was significantly increased. Inflammatory markers	
		ROS, TNF-α, IL-10 met +3 J/cm^2^ significantly better	
Diode LASER -660 nm (InGaAIP)	30mW, single dose of 1 J/cm^2^, CW	Greater cell metabolic activity in irradiated group compared to control in 24 and 48 h. Higher density of viable cells in the LASER group	Costa et al. (2021)
High intensity red LED—600–700 nm	400 mW/cm^2^, 2, 4, 6, 8, and 10 J/cm2 for 5, 10, 15, 20, and 25 s continuous output	High-intensity red LED inhibits production of pro-inflammatory cytokines in hPDLSCs induced by TNF-α via encouraging the synthesis of ATP thereby promoting regeneration	(Yamauchi et al., 2018)
Near infrared diode LASERs - 810 or 940 nm	Energy density of 0.5, 1.5 and 2.5 J/cm^2^; 100 mW	Increase in viability was observed only with 940 nm LASER irradiation at energy density of 2.5 J/cm^2^. Cell proliferation significantly increased with 940 nm LASER irradiation energy density of 2.5 J/cm^2^ at all the time points compared to other groups	Rigi-Ladez et al. (2022)
LASERs and LEDs used within the 630–1064 nm wavelength range	245 studies assessed out of which 11 met the inclusion criteria	No agreement among scientists on PBM methods. Wavelengths between 630 and 830 nm produced beneficial results, the use of a near-infrared (NIR) wavelength at 940 nm may not	Mylona et al. (2022)
940 nm Diode LASER	100mW, CW, 4 J/cm^2^, 3 sessions at every 48 h	Proliferation: No significant differences between test and control	Gholami et al. (2022a)
		Osteogenesis: after 14th and 21st day, test groups showed greater mineralised tissue formation in comparison to test group	
LASER	250mW, 20 s, 2, 4, 6 and 8 J/cm^2^ vs. control 0 J/cm^2^, perpendicular, scanning mode, every other day	Proliferation	Wang et al. (2022)
Nd:YAG 1064 nm		7th day - 4, 6, 8 J/cm^2^ significantly better with highest activity at 6 J/cm^2^ Osteogenesis	
		2–6 J/cm^2^ - ideal	
		8 J/cm^2^—osteogenesis is supressed	
LASER at 635, 660, 808 and 980 nm	LASER light with energies of 1, 1.5, 2.5, and 4 J	Proliferation	Etemadi et al. (2022)
		PDLSCs stimulated by PBM of 635, 660, 808 and 980 nm. Highest cell survival was seen after being exposed to a 980 nm LASER with an energy density of 4 J cm^2^ on day 3	
940 nm	940 nm (NIR)	Proliferation: No significant differences between LASER and control group on 3rd day	Gholami et al. (2022a)
And 660 nm (red) LASERs	100mW, CW	Osteogenesis: No significant differences between LASER	
	400 µm tip, 4	and control group on 14th and 21st day	
	J/cm 2, pulsed mode compared to 660 nm (red) irradiations at 3 J/cm 2	Compared to 660 nm (red) irradiation, this impact was stronger at 940 nm (NIR)	

FOOTNOTE—RUN, X2- Runt-related transcription factor 2; OCN, Osteoclacin; ALP-Alkaline Phosphatase; NIR-near-infra-red; HPDLSC- human periodontal ligament stem cell; CW- continuous wave; LED- light emitting diode; HFLPLI- High-fluence low-power LASER, irradiation.

Literature evidence shows that LASER PBM on PDLSCs significantly increases proliferation and osteogenesis. However, majority of studies show wide variation in the wavelength and energy settings for bio stimulation, and this difference in the amount of power and time settings makes the stem cell proliferation vary (Felician et al., 2023). Initially Soares et al. and Wu et al. have shown there is significant increase in proliferation when the power of 660 nm red diode LASER is increased from 1 to 5 J/cm^2^ and also when duration of exposure is increased. However, it has been noted that increasing the energy setting or duration of exposure doesn’t change the rate of proliferation or osteogenic gene expression (Gholami et al., 2022a; Gholami et al., 2022b). This signifies that energy settings, time of irradiation and follow up with subsequent irradiations makes difference when the optimal energy setting is applied. Still, there is no standardized protocol to stimulate PDSLCs to the maximum.

It was observed that using 600–700 nm LED in the power of 8 J/cm^2^ had the highest rate of proliferation and osteogenic differentiation of PDLSCs and increasing the duration to 3 weeks also proved to be optimal in maintaining regeneration capability (Yamauchi et al., 2018). This suggests that lower the wavelength of light source higher is the photo bio modulatory property.

Further, Gholami et al. compared 940 nm diode and 660 nm (red LASER) and showed that group receiving 940 nm irradiation showed better cell proliferation and differentiation on day 3 and also 3 weeks of LASER application. Similarly, Chaweewannakorn et al. showed that by using three different wavelengths of LEDs, there is a decline in proliferation on day 8th of irradiated groups and stated that inadequate wavelength can cause damage to cell viability of PDLSCs thereby emphasizing the importance of optimal wavelength. Rigi Ladez et al. stated that using a wavelength of diode LASER of 940 nm instead of 810 nm showed a better proliferation of PDLSCs which is in concurrence of Gholami et al. Wang et al. and Etemadi et al. who observed positive effects of LASER irradiation on PDLSCs on day 21 and day 5 respectively.

LASER PBM is a beneficial technique for tissue regeneration even in inflammatory regions and has a substantial anti-inflammatory impact by lowering pro-inflammatory cytokines (Yamauchi et al., 2018). In a systematic review on the effect of LASERs and LEDs on PDLSC proliferation by Mylona et al., it was observed that the stemness and differentiation abilities of periodontal ligament stem cells can be improved by photo biomodulation. On PBM techniques, such as duration, wavelength and energy settings, there is currently no consensus among experts. The usage of a near-infrared (NIR) wavelength at 940 nm may not have the same positive effects as wavelengths between 630 nm and 810 nm. It was said that the fluence shouldn’t be greater than 8 J/cm2 when utilizing LED therapy devices and that it shouldn’t be greater than 4 J/cm2 while using LASERs.

## Conclusion

Photo-biomodulation, a property which stimulates PDLSCs and other stem cells in general, is a property specially owned by cold LASERs. With the above-mentioned evidences, it can be noted that by decreasing the wavelength of the LASER, better PBM can be achieved; however, there are various other factors such the spot size, time and mode of irradiation which decides optimal stimulation of PDLSCs. In the future, it can be seen that post periodontal treatments with Erbium group of LASERs which operate at a higher wavelength may still require a LASER of lower wavelength for bio-modulation, thereby giving rise to a dual LASER therapy. The above-mentioned studies in the table hold a lot of ambiguity as different types of LASERs are used with different wavelengths and power settings as studies are mostly done by individual research scholars often funded by commercial LASER companies. Hence, it is necessary for the governing bodies around the globe to come up with a consensus and a protocol to make the most of the stimulation of PDLSCs.
